# Monkeypox Virus Clade IIa Infections, Liberia, 2023–2024

**DOI:** 10.3201/eid3109.250271

**Published:** 2025-09

**Authors:** Dougbeh Chris Nyan, Irina Maljkovic Berry, Bode I. Shobayo, Monika Mehta, Gabriella Worwa, Shawn Hirsch, Sarah E. Klim, Fahn Taweh, Kalilu S. Donzo, B.M. Freeman, Alberta B. Corvah, Francis Jaryan, Julius S.M. Gilayeneh, Ian Crozier, Courtney Renken, Laura McNay, J. Soka Moses, Jens H. Kuhn, Lisa E. Hensley, Sara E. Zufan

**Affiliations:** National Public Health Institute of Liberia, Monrovia, Liberia (D.C. Nyan, B.I. Shobayo, F. Taweh, K.S. Donzo, B.M. Freeman, A.B. Corvah, F. Jaryan, J.S.M. Gilayeneh, J.S. Moses); National Institutes of Health, Frederick, Maryland, USA (I. Maljkovic Berry, M. Mehta, G. Worwa, S. Hirsch, S.E. Klim, J.H. Kuhn, S.E. Zufan); Clinical Monitoring Research Program Directorate, Frederick National Laboratory for Cancer Research, Frederick (I. Crozier); National Institutes of Health, Bethesda, Maryland, USA (C. Renken, L. McNay); US Department of Agriculture, Manhattan, Kansas, USA (L.E. Hensley)

**Keywords:** monkeypox virus, MPXV, viruses, mpox, emerging infections, zoonoses, zoonotic diseases, Liberia

## Abstract

We performed monkeypox virus genome sequencing on clinical samples from Liberia, yielding 5 clade IIa genomes. Our analysis found no evidence of sustained human-to-human transmission, suggesting independent zoonotic spillovers from a diverse viral lineage. Public health officials should continue monitoring and sequencing efforts to identify emerging monkeypox virus lineages.

Monkeypox virus (MPXV; Poxviridae: *Orthopoxvirus monkeypox*) isolates cluster into 2 major clades, I and II, and each has subclades a and b (*1*,*2*). Clades Ia and IIa, primarily circulating in Equatorial Africa, generally cause zoonotic spillovers, whereas specific lineages of clades Ib and IIb from the 2024 outbreak in Central Africa have been associated with sustained human-to-human transmission (*3*,*4*). Clade I typically causes more severe disease and higher case-fatality rates than clade II, and the 2024 outbreaks in Democratic Republic of the Congo showed lower case-fatality rates for clade Ia and less-severe clade Ib infections (*5*). Those clade Ib–driven outbreaks nonetheless include severe cases, underscoring the need to elucidate genetic determinants of virulence within and between MPXV lineages.

During December 2023–August 2024, we collected 41 clinical samples (lesion swabs, crusts, whole blood, and serum) from 21 persons in Liberia suspected to have mpox. We recorded epidemiologic and clinical data on a standardized form based on the Integrated Disease Surveillance and Response Technical Guidelines (https://www.who.int/publications/i/item/WHO-AF-WHE-CPI-05-2019#:~:text=The%20third%20edition%20of%20the%20Integrated%20Disease%20Surveillance,and%20the%20U.S.%20Agency%20for%20International%20Development%20%28USAID%29). In August 2024, an mpox outbreak caused by clade Ib MPXV was declared a public health emergency of international concern (*6*), whereas clade IIb continued to circulate globally. Diagnostic testing at the National Public Health Institute of Liberia National Reference Laboratory (https://nphil.gov.lr) used an MPXV real-time PCR (Liferiver Bio-Tech Corp., http://www.liferiverbiotech.com) for initial virus detection and confirmation (Table) (*7*).

**Table:** Patient and sample characteristics from suspected mpox cases in monkeypox virus clade IIa infections, Liberia, 2023–2024*

Sample ID	Age, y/sex	Location	Symptom onset date	Sample collection date	Liferiver MPXV result	Liferiver MPXV (FAM) Ct
*LIB-MPV24-001*	22/F	Nimba	2023 Dec 28	2024 Jan 9	+	36.74
*LIB-MPV24-007*	5/M	Sinoe	2024 Feb 8	2024 Feb 12	+	27.92
*LIB-MPV24-014*	30/M	Nimba	2024 Mar 8	2024 Mar 12	+	37.06
LIB-MPV24-015	39/F	Nimba	2024 Mar 10	2024 Mar 11	+	37.56
LIB-MPV24-027	24/F	Grand Kru	2024 May 2	2024 May 6	+	37.63
*LIB-MPV24-037*	7/M	Nimba	2024 Jun 26	2024 Jun 4	+	34.27
**LIB-MPV24-054**	7/F	Sinoe	2024 Aug 20	2024 Aug 24	+	29.98
LIB-MPV24-069	2/M	Lofa	2024 Aug 27	2024 Aug 29	+	30.33
*LIB-MPV24-087*	27/F	Sinoe	2024 Sep 4	2024 Sep 5	+	26.44
**LIB-MPV24-107**	6/M	River Gee	2024 Aug 17	2024 Sep 7	+	36.8
LIB-MPV24-114	20/M	Bong	2024 Sep 3	2024 Sep 11	−	38.5
LIB-MPV24-116	16/M	Bong	2024 Sep 4	2024 Sep 11	−	Undetermined
LIB-MPV24-117	12/M	Lofa	2024 Sep 3	2024 Sep 9	−	Undetermined
LIB-MPV24-118	16/F	Grand Gedeh	2024 Aug 25	2024 Sep 10	−	Undetermined
LIB-MPV24-119	56/M	Montserrado	2024 Sep 10	2024 Sep 13	−	Undetermined
**LIB-MPV24-123**	29/M	Bong	2024 Sep 3	2024 Sep 14	−	Undetermined
**LIB-MPV24-136**	28/M	Montserrado	2024 Aug 31	2024 Sep 16	−	Undetermined
LIB-MPV24-141	23/M	Sinoe	2024 Sep 10	2024 Sep 13	−	Undetermined
LIB-MPV24-142	17/F	Grand Gedeh	2024 Aug 24	2024 Sep 14	−	Undetermined
LIB-MPV24-143	58/F	Grand Kru	2024 Aug 30	2024 Sep 13	−	Undetermined
LIB-MPV24-144	1/M	Sinoe	2024 Sep 10	2024 Sep 13	−	Undetermined

*Sample IDs in italics yielded near complete genomes (>98%), and sample IDs in bold met the threshold for detectable MPXV reads, defined as >1 read per kilobase of the MPXV reference genome per 1 million filtered reads. Of the 5 PCR-positive samples reported by the National Public Health Institute of Liberia that did not yield sequences, 3 had undetectable MPXV reads and insufficient DNA input (<10  ng), and 2 had detectable reads but lacked sufficient coverage for consensus genome generation. +, positive; −, negative; Ct, cycle threshold; FAM, fluorescein; MPXV, monkeypox virus.

To determine which MPXV clades were circulating in Liberia, the National Public Health Institute of Liberia partnered with the Integrated Research Facility at Fort Detrick (IRF-Frederick) to perform genomic sequencing. That collaboration aimed to identify previously undetected MPXV strains and possible co-infections to inform public health measures and clinical management.

We transferred 41 inactivated specimens from 21 patients to IRF-Frederick, which extracted nucleic acids by using a MagMAX Viral/Pathogen Nucleic Acid Isolation Kit (Thermo Fisher Scientific, https://www.thermofisher.com) on a KingFisher Flex system (Thermo Fisher Scientific). We prepared sequencing libraries by using the DNA Prep with Enrichment kit with 10- to 49-ng input (Illumina, https://www.illumina.com) and enriched the libraries by using the Comprehensive Viral Research Panel (Twist Biosciences, https://www.twistbioscience.com). We prepared the libraries by using unique dual indices (IDT for Illumina UDIs, Set A; Illumina) followed by precapture pooling assembled in 6-plex with up to 500 ng per library. After capture, we pooled all libraries equimolarly and loaded the libraries on a single NextSeq 1000/2000 P1 XLEAP-SBS 300-cycle flow cell (2 × 150 bp; Illumina). This enrichment-based metagenomic sequencing approach enabled detection of MPXV and other viral DNA pathogens present in the samples.

We analyzed sequencing reads with EsViritu version 0.2.3 (https://github.com/cmmr/EsViritu) to identify viruses and quantify viral genomes. We further processed MPXV reads by using the nf-core/viralrecon pipeline version 2.6.0 (https://nf-co.re/viralrecon/2.6.0/) and aligned the reads to a reference MPXV strain rom GenBank (accession no. KJ642613.1).

Overall, we detected MPXV DNA in 10 patients and varicella zoster virus (VZV) in 10 patients, including 2 cases of possible MPXV–VZV co-infection (Appendix Figure 1). We also detected partial genomes of Epstein-Barr virus (Herpesviridae: *Lymphocryptovirus humangamma 4*), hepatitis B virus (Hepadnaviridae: *Orthohepadnavirus hominoidei*), and torque teno mini virus 8 (Anelloviridae: *Betatorquevirus homini 8*). Detection of multiple viral pathogens, including VZV and MPXV within the same time frame, reinforces the value of broad metagenomic approaches for diagnosing lesions of unknown etiology, particularly when clinical manifestations overlap.

We assembled 5 near-complete (>98%) MPXV genomes; Nextclade (https://docs.nextstrain.org/projects/nextclade/en/stable/index.html) analysis confirmed that all belonged to clade IIa. We deposited all sequences in GenBank (accession nos. PV122071–5). To determine whether those MPXV cases arose from zoonotic spillover or ongoing human-to-human transmission, we analyzed phylogenetic relationships, mutation rates, and apolipoprotein B mRNA editing enzyme, catalytic subunit 3G (APOBEC3)–mediated editing patterns (Figure; Appendix Figure 2). Maximum-likelihood analysis and APOBEC3 ancestral reconstruction using squirrel version 1.0.11 (https://github.com/aineniamh/squirrel) showed limited APOBEC3-mediated editing (6/61 internal single-nucleotide polymorphisms [9.8%]). By contrast, clade IIb viruses from the 2022 global outbreak exhibited extensive APOBEC3-driven hypermutation (*8*).

**Figure:**
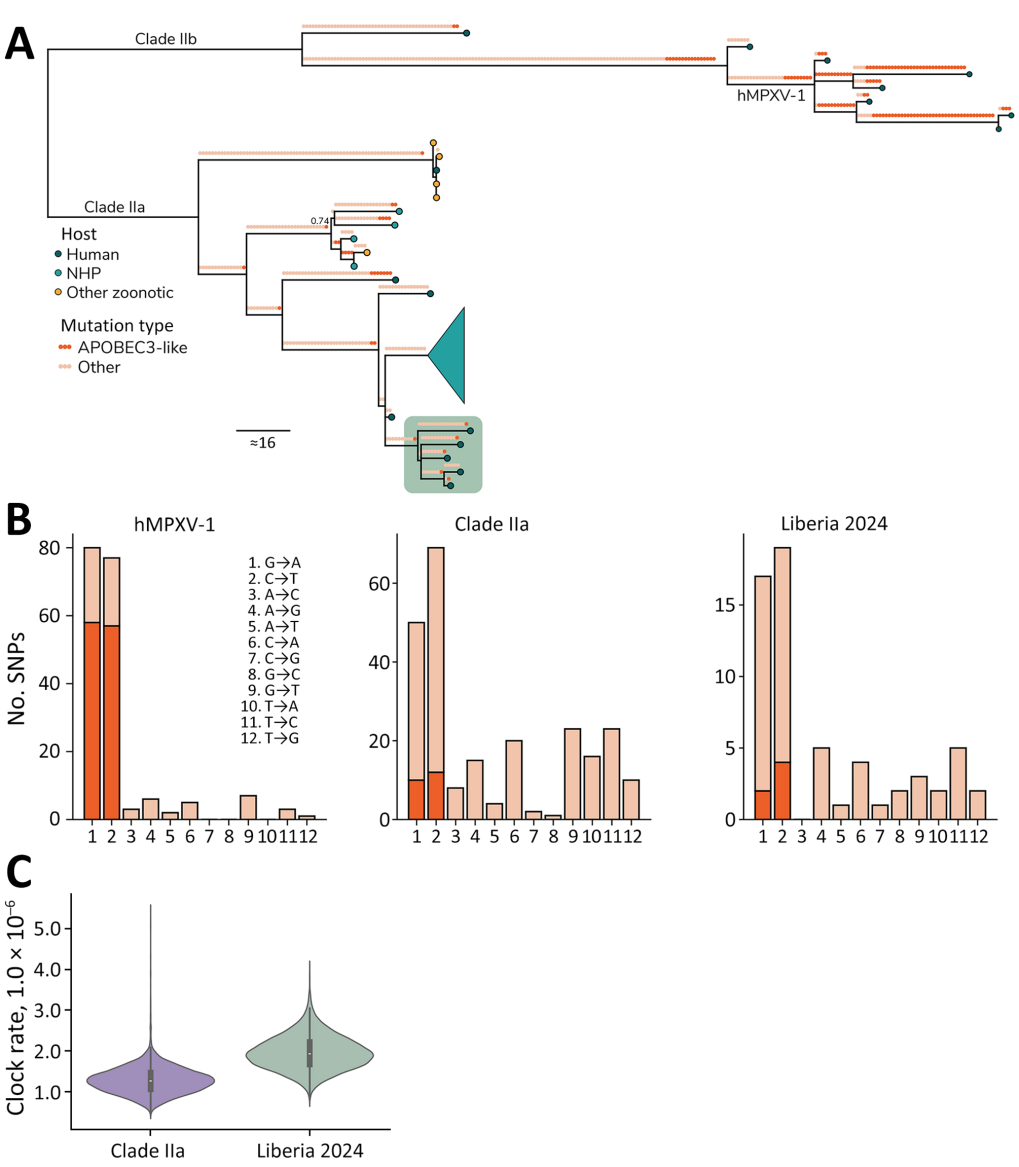
Sequencing characteristics of monkeypox virus clade IIa infections, Liberia, 2023–2024. A) Ancestral state reconstruction of the clade II phylogeny comparing the 5 new sequences against all publicly available clade IIa genomes (n = 27) and a subset of clade IIb sequences for context (n = 7), including hMPXV-1. Mutations are annotated along each branch with circles colored by whether it is an APOBEC3-like signature (TC→TT and GA→AA; dark orange) or whether it is another mutation type (light orange). Branch tips show circles colored by host (human, dark blue; NHP, turquoise; other, yellow). The sequences generated from this study are displayed in the green box. A large clade from Côte d'Ivoire NHPs is collapsed (turquoise funnel). Ultrafast bootstrap support is indicated only at nodes for which support <0.75. B) Number of APOBEC3-like SNPs of all mutations for subclade hMPXV-1, prior clade IIa sequences, and the new clade IIa found in Liberia. C) Comparison of the evolutionary clock rate of the prior clade IIa and new Liberian clade IIa sequences estimated under the local clock model. APOBEC3, apolipoprotein B mRNA editing enzyme catalytic subunit 3; hMPXV-1, sustained human monkeypox virus outbreak; NHP, nonhuman primate; SNP, single-nucleotide polymorphism.

Next, by using a fixed local clock model in BEAST version 1.10.5 (https://beast.community), we estimated a mean evolutionary rate of 1.96 × 10^−6^ substitutions/site/year (95% highest posterior probability 7.61 × 10^−7^ to 3.92 × 10^−6^) for the clade IIa sequences. That rate is ≈15-fold lower than the APOBEC3-driven rates reported for 2022 clade IIb strains (*9*), further indicating that those infections reflect spillover events rather than sustained human-to-human transmission.

Finally, because MPXV diversity is largely shaped by geographic separation rather than time (*10*), the distinct phylogenetic clustering of cases from Sinoe County versus those from Nimba County (Appendix Figure 2) also supports independent zoonotic spillover events rather than a single transmission chain. However, inference is limited by the small number of available sequences.

In conclusion, this study contributes MPXV genomes from Liberia, addressing a multidecade absence of genomic data from human cases in western Africa. Continued monitoring and sequencing efforts are essential for identifying emerging virus lineages, informing targeted public health interventions, and guiding clinical management strategies to address the varied presentations associated with different MPXV clades.

Appendix Additional information for monkeypox virus clade IIa infections, Liberia, 2023–2024.
