# Supplementary material for: Monkeypox Virus Clade IIa Infections, Liberia, 2023–2024
Source: Emerg Infect Dis. 2025 Sep;31(9):1848–51. doi: 10.3201/eid3109.250271 (PMC12407201; doi:10.3201/eid3109.250271)
Supplement: Appendix — Additional information for monkeypox virus clade IIa infections, Liberia, 2023–2024. [file 25-0271-Techapp-s1.pdf]

Article DOI: https://doi.org/10.3201/eid3109.250271

EID cannot ensure accessibility for supplementary materials supplied by authors. Readers who have difficulty accessing supplementary content should contact the authors for assistance.

Monkeypox Virus Clade IIa Infections, Liberia, 2023–2024

Appendix

Appendix Table. Metadata and alignment inclusion for clade II MPXV genomes used in phylogenetic and phylodynamic analyses*

					Alignment	
Accession	Host	Country	Collection date	Clade	ML	BEAST
KJ642613	Human	DRC	1970–09–01	IIa	Yes	Yes
DQ011156	Human	Liberia	1970–10–02	IIa	Yes	Yes
AY741551	Human	Sierra Leone	1970–11–26	IIa	Yes	Yes
KP849470	Human	Côte d'Ivoire	1971–10–18	IIa	Yes	Yes
MT903346	Gambian rat	USA	2003–05–03	IIa	Yes	Yes
MT724769	Swamp rat	DRC	2012–01–01	IIa	Yes	Yes
KJ136820	Sooty monkey	Côte d'Ivoire	2012–03	IIa	Yes	Yes
AY603973	Macaque	USA	1961	IIa	Yes	Yes
AY753185	Macaque	Denmark	1958–06–30	IIa	Yes	Yes
DQ01153	Prairie dog	USA	2003–05–13	IIa	Yes	Yes
DQ011157	Human	USA	2003–05–24	IIa	Yes	Yes
KJ642614	Orangutan	Netherlands	1965–12–31	IIa	Yes	Yes
KJ642616	Chimp	France	1968	IIa	Yes	Yes
MN346691	Environment	Côte d'Ivoire	2017–03–11	IIa	Yes	Yes
MN346692	Chimp	Côte d'Ivoire	2017–03–05	IIa	Yes	Yes
MN346693	Chimp	Côte d'Ivoire	2017–04–08	IIa	Yes	Yes
MN346694	Chimp	Côte d'Ivoire	2017–03–28	IIa	Yes	Yes
MN346695	Chimp	Côte d'Ivoire	2017–04–02	IIa	Yes	Yes
MN346696	Chimp	Côte d'Ivoire	2017–04–03	IIa	Yes	Yes
MN346697	Chimp	Côte d'Ivoire	2017–04–14	IIa	Yes	Yes
MN346698	Chimp	Côte d'Ivoire	2017–01–24	IIa	Yes	Yes
MN346699	Chimp	Côte d'Ivoire	2017–01–23	IIa	Yes	Yes
MN346700	Chimp	Côte d'Ivoire	2017–01–31	IIa	Yes	Yes
MN346701	Chimp	Côte d'Ivoire	2017–01–14	IIa	Yes	Yes
MN346702	Chimp	Côte d'Ivoire	2018–05–11	IIa	Yes	Yes
MN346703	Chimp	Côte d'Ivoire	2018–05–20	IIa	Yes	Yes
MT903347	Dormouse	USA	2003–04–21	IIa	Yes	Yes
MT903348	Rope squirrel	USA	2003–04–21	IIa	Yes	Yes
KJ642615	Human	Nigeria	1978	IIb	Yes	No
KJ642617	Human	Nigeria	1971–04–14	IIb	Yes	No
MK783027	Human	Nigeria	2017–11–09	IIb	Yes	No
MK783033	Human	Nigeria	2017–10–09	IIb	Yes	No
MN648051	Human	Israel	2017–10–04	IIb	Yes	No
ON563414	Human	USA	2022–05–19	IIb	Yes	No
OP413718	Human	UK	2022–08	IIb	Yes	No
OP415257	Human	UK	2022–08	IIb	Yes	No

*Metadata include host, country of origin, collection date, and assigned clade (IIa or IIb). The final 2 columns indicate whether each sequence was included in the maximum likelihood and BEAST alignments used for ancestral state reconstruction and time-resolved phylogenetic analyses, respectively. Collection dates are shown in YYYY–MM–DD format where available. ML, maximum likelihood; MPXV, monkeypox virus

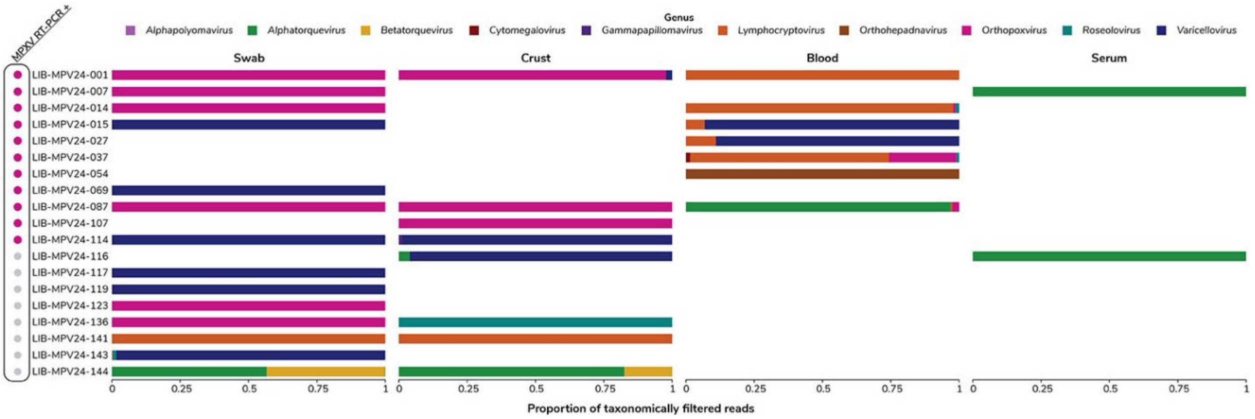

Appendix Figure 1. Composition of viral genera relative abundance of mpox suspected patient specimens. Relative abundance of viral genera after taxonomic filtration using EsViritu (v0.2.3). Empty panels indicate no sample available for testing.

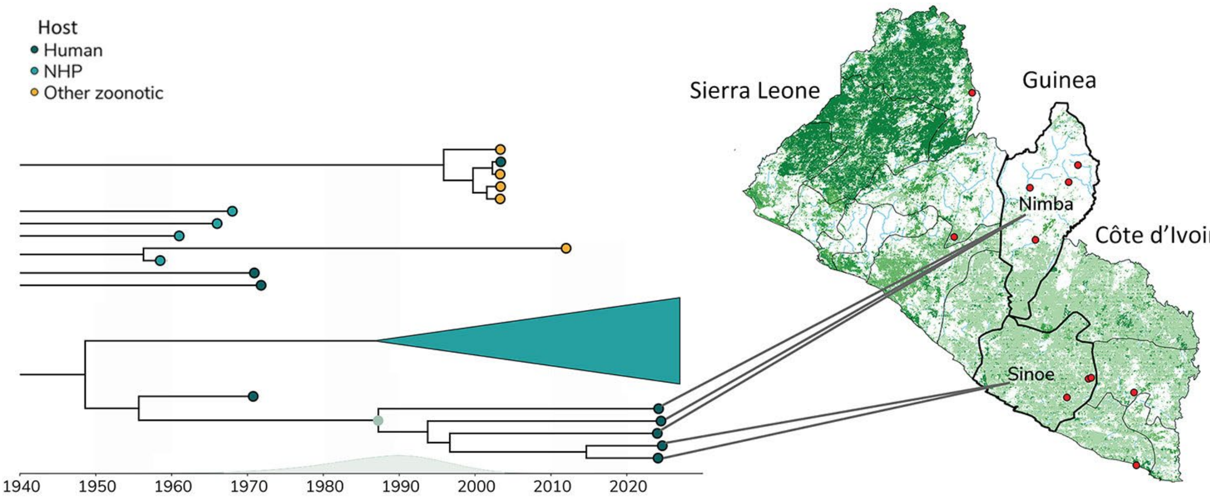

Appendix Figure 2. Time-resolved phylogeny of clade IIa. The Côte d'Ivoire NHP clade is collapsed (turquoise funnel). The clade of sequences generated in this study is annotated with a green circle at the node with the associated distribution of the tMRCA on the *x*-axis. Gray lines connect each sequence on the phylogeny to its corresponding location on the map of Liberia, which displays rivers, forest cover, and county-level attribution. Filled red circles denote approximate collection sites of PCR-positive specimens. Accession numbers used for phylogenetic and phylodynamic analyses are in the Appendix Table.
